# Right ventricular lead sensing latency in pacemaker therapy

**DOI:** 10.1002/joa3.12767

**Published:** 2022-08-23

**Authors:** Fani Zagkli, Nikoleta Kalovrenti, Panagiotis Patrinos, Panagiotis Chronopoulos, John Chiladakis

**Affiliations:** ^1^ Department of Cardiology University Hospital of Patras Patras Greece

**Keywords:** pacemaker, pacing system analyzer, right ventricular lead, sensing latency

## Abstract

**Background:**

Pacemaker implantation involves intraoperative testing of ventricular sensing using a device called a pacing system analyzer (PSA). The value obtained is expected to correspond to those taken by the pacemaker after its implantation. This study determined the latency period for sensing intracardiac electrogram (EGM) by the right ventricular (RV) lead.

**Methods:**

Patients without significant heart disease and underlying intrinsic atrioventricular (AV) conduction underwent Medtronic or Abbott dual‐chamber pacemaker implantation with the RV lead positioned on the mid‐septum. Real‐time sensing data were obtained through PSA and after pacemaker implantation to evaluate latency as the time interval Q‐VS between the onset of QRS on surface electrocardiogram and the sensed EGM by the RV lead.

**Results:**

Of 157 patients, 105 had narrow QRS (<120 ms) and 52 had wide QRS of complete right bundle branch block (RBBB). Both narrow‐QRS and RBBB patients had longer sensing latency through PSA (50.9 ± 24.2 and 67.8 ± 32.9 ms, respectively) than through pacemaker (18.2 ± 12.8 and 31.2 ± 14.8 ms, respectively, both *p* < 0.001). RBBB patients had longer sensing latency compared with narrow QRS patients, either through PSA or through pacemaker (*p* < 0.001). The sensing latency of Medtronic recipients was longer than those of Abbott in narrow‐QRS (*p < 0*.05), but not in RBBB.

**Conclusion:**

We demonstrated longer RV lead sensing latency (1) through PSA than through pacemaker, (2) in RBBB than in narrow‐QRS, and (3) in Medtronic pacemakers compared with Abbott pacemakers. Knowledge of sensing latency helps the optimization of the AV delay.

## 1 INTRODUCTION

Sensing intrinsic ventricular activity is a basic element in pacing. The pacemaker recognizes the heart's own intrinsic signal through the implanted right ventricular (RV) lead by analyzing the local intracardiac electrogram (EGM). Once the RV lead is in place, intraoperative measurements include the determination of the sensing threshold using an external or inbuilt programmer pacing system analyzer (PSA) unit.^1^ The PSA application displays real‐time signals for surface electrocardiogram (ECG), generates sensing markers for local EGM, and outputs those signals to a recorder as well. Following implantation, the implanted pulse generator is expected to replicate the sensing data obtained during the PSA operation. Since both the PSA and the pacemaker use the EGM for sensing analysis with specific reference to the QRS on the ECG as read by electrodes placed on the skin's surface, timely recognition of QRS is a preliminary step for proper sensing function.

The different methods of recording the electrical activity of the heart, i.e. ECG and EGM, raise the question as to whether the two signals coincide in time, in the knowledge that any signal should be expected to be displayed after a certain gap of time because of propagation and processing delays. In this context, clinical interest has grown substantially in the field of cardiac resynchronization therapy (CRT) with several investigators reporting differences in timing between the QRS on the ECG before the sensed EGM on the left ventricular lead.^2^, ^3^, ^4^ A more recent report merits consideration as to the observation of significant electrical delay in CRT recipients by using the PSA module of the Medtronic 2090 programmer.^5^ The proof of sensing latency may have far‐reaching implications concerning the management of intrinsic ventricular activity or even the occurrence of unnecessary ventricular pacing as a result of fusion/pseudofusion, a common pitfall in device follow‐up, since not only pacemakers but also cardioverter‐defibrillators and CRT devices use the RV lead itself for sensing.^6^, ^7^, ^8^, ^9^

Still, basic knowledge regarding timing relationships between QRS and acquired EGM from the RV lead is lacking. In this work, the RV lead sensing latency refers to the gap of time by which the ventricular sensed EGM gets delayed relative to the QRS on ECG. It was the specific aim of our study to determine the RV lead sensing latency in patients without significant heart disease and intrinsic atrioventricular (AV) conduction, undergoing dual‐chamber pacemaker implantation of different manufacturers (Medtronic Inc. or Abbott [formerly St Jude Medical, St Paul, MN]). Furthermore, we clarified by intra‐individual comparisons whether the intraoperatively RV electrical delay data through PSA correspond to those obtained through the implanted pacemaker.

## 2 METHODS

### 2.1 Patients

We studied 157 patients (mean age: 76 ± 9 years, range: 58–94 years) without evidence of significant heart disease who underwent common dual‐chamber pacemaker implantation for standard indication following written, informed consent. Patients were considered for inclusion in the study if they were hemodynamically stable and asymptomatic during operation and had intrinsic AV conduction of more than 40 beats per minute because of sinus bradycardia or 2:1 AV block with constant PR intervals. Eligible patients did not have a history of organic heart disease or heart failure and had normal left ventricular dimension and systolic ejection fraction ≥50%, as assessed by two‐dimensional echocardiography. Patients were excluded if they had: reversible causes for AV block, atrial or ventricular arrhythmia, acute coronary syndrome and/or unstable angina, severe pulmonary disease, or renal insufficiency; if they needed sympathomimetic drugs or atropine or temporary pacing during operation, as those undergoing pulse generator change or lead revision.

The patient population was divided into two groups: narrow QRS (<120 ms) or wide QRS (≥120 ms) *according to their* baseline *QRS duration* on 12‐lead ECG before pacemaker implantation.

### 2.2 Implantation procedure

All patients underwent their first, Medtronic, or Abbott, transvenous dual‐chamber pacemaker implantation under continuous ECG monitoring through an external surface 12‐lead ECG polygraph (WorkMate Claris™, St. Jude). Standard implant techniques were used with local anesthesia. The electrode leads were introduced via the cephalic and/or the subclavian vein, and the pulse generator was placed in a subpectoral pocket. Pacemaker devices included the Medtronic models Adapta™ and Attesta MRI™, and the Abbott models Victory™, Endurity™, Assurity MRI™, and Ensura MRI™. The same bipolar steroid‐eluting active fixation RV pacing lead of the same brand (Medtronic 4076, tip electrode surface area 4.2 mm^2^ or Abbott 2088TC, tip electrode surface area 6.9 mm^2^, both of 58 cm length with tip to ring spacing 10 mm), was used for RV mid‐septal placement. The optimal RV lead position was confirmed by combined fluoroscopy views and characteristic paced ECG criteria.^10^, ^11^

### 2.3 Electrical testing

*Programmers* screens (Medtronic CareLink 2090 with 2290 analyzer or Abbott Merlin™ PCS) were configured to display in real‐time simultaneous ECG lead II, markers, and RV EGM. The standard programmer's ECG cables and lead wires of Medtronic and Abbott manufacturers (5.4 and 4 m in length, respectively), connected the programmers to skin electrodes for ECG. The use of a PSA unit (Medtronic module 2290 and Abbott mode**l** EX3100) of the same pacemaker brand served for EGM, peak‐to‐peak R‐wave amplitude, and maximum voltage deflection/time (slew rate) measurements. Note that in order to obtain optimal results, each pacemaker manufacturer supplies PSAs with band‐pass filters that match the filters used in their pacemaker systems.^1^ PSA cable connections (Medtronic model 5833SL of 3.66 m length and St. Jude Medical model 4051 L of 3.7 m length, respectively) were used to display the EGM through the implanted RV lead in bipolar mode by using crocodile clips on the lead terminal pins. Sensing measurements were taken through PSA after helix extension of the active fixation RV lead. Immediately after completion of the implant procedure, manual RV lead sensing testing was repeated via a programmerʼs telemetry wand placed over the pacemaker. PSA and pacemaker sensing were tested at a similar stable spontaneous heart rate by temporarily programming the devices to the VVI mode at a rate below the patient's intrinsic heart rate. For each patient, the PSA of the same pacemaker brand was used.

### 2.4 ECG analysis

**FIGURE 1:**
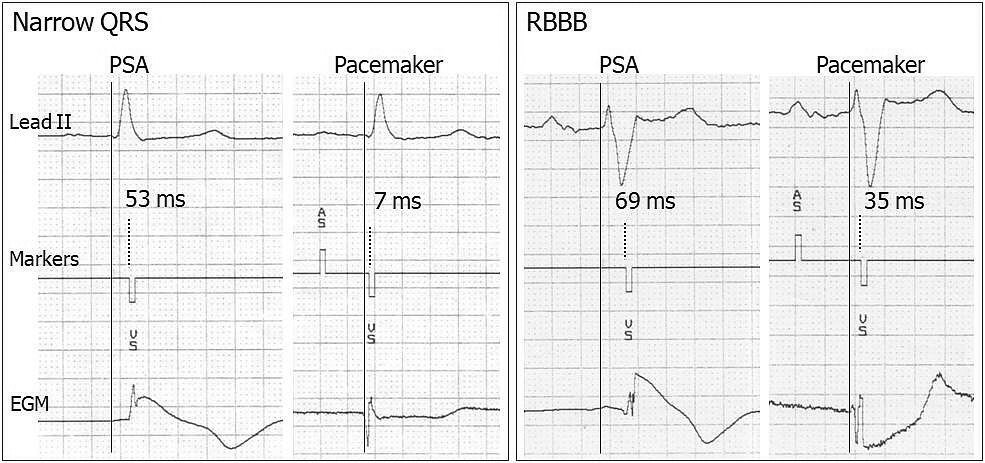
Examples of RV lead sensing latency by the use of Medtronic devices. Real‐time electrograms were retrieved through PSA and pacemaker in narrow QRS (left panel) and RBBB (right panel) at a sweep speed of 50 mm/s. The calipers are aligned with the onset of the QRS complex in lead II (continuous vertical line) and the onset of VS marker (dotted vertical line). Note the shorter RV lead sensing latency through pacemaker compared with PSA in both narrow QRS and RBBB and the prolonged RV lead sensing latency in the presence of RBBB. AS, atrial sensed event; PSA, pacing system analyser; RBBB, right bundle branch block; RV, right ventricular; VS, ventricular sensed event.

**FIGURE 2:**
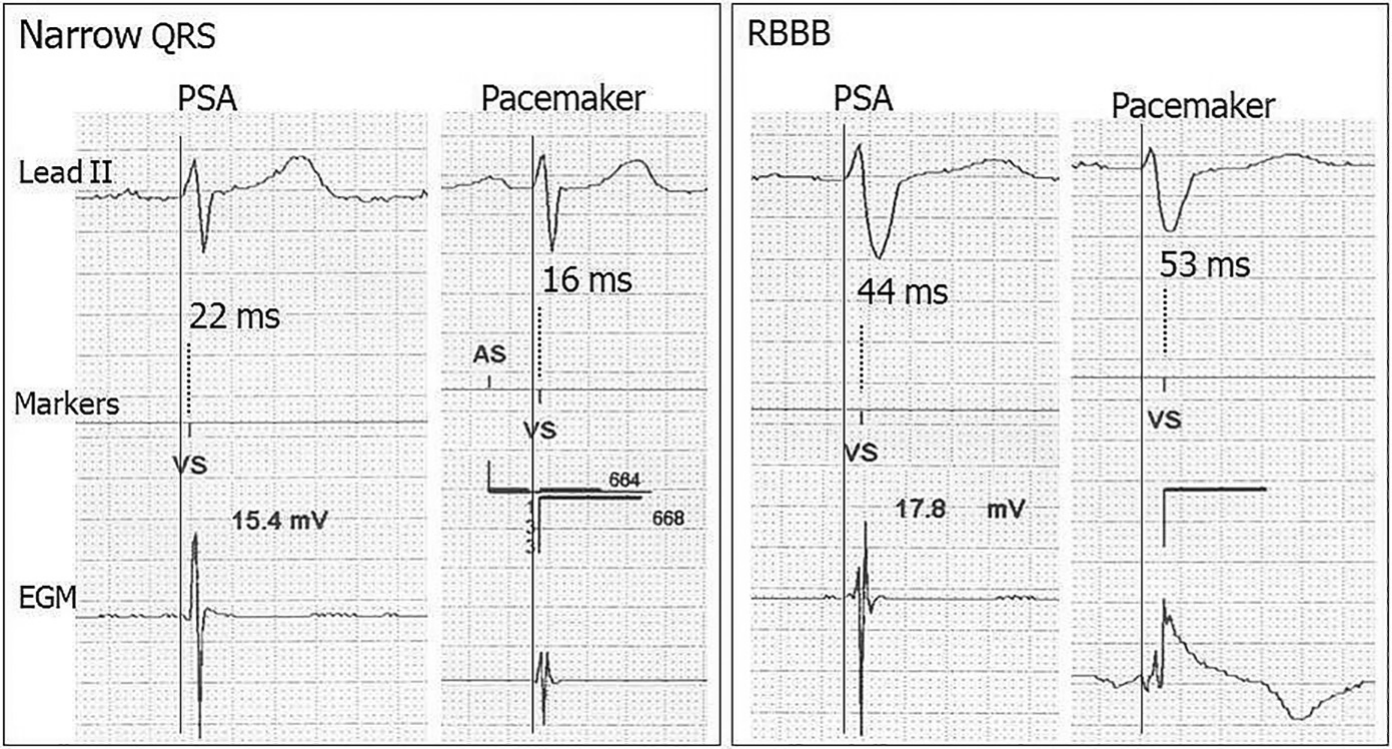
Examples of RV lead sensing latency by the use of Abbott devices In Narrow‐QRS (left panel) and RBBB (right panel). Note the prolonged RV lead sensing latency in RBBB compared with narrow QRS. Here, in the presence of RBBB, the RV lead sensing latency is slightly longer through the pacemaker compared with PSA. Same abbreviations as described in Figure 1.

A 12‐lead intrinsic rhythm baseline ECG was recorded before implantation at a paper speed of 50 mm/s with 10 mV/cm gain. The QRS duration was measured as the interval from the first onset of QRS in any lead until the latest offset in any lead. Wide QRS morphology was classified according to World Health Organization criteria.^12^ Real‐time rhythm strips were obtained through the programmer's internal printer during the PSA session and at immediate pacemaker implantation comprising concurrent ECG lead II, marker telemetry, and EGM at a sweep speed of 50 mm/sec. The RV lead sensing latency was defined as the time delay Q‐VS between the onset of QRS on ECG and the time at which the EGM was sensed, identified as a released ventricular sensed (“VS”) event marker by the device (Figures 1 and 2). Baseline ECG and rhythm strip data were analyzed manually over three cardiac cycles and then averaged using an electronic digitizer (Yansen Digital Caliper, Central Tools, Inc) by a single investigator, with an intraobserver variability of 0.83 ± 5.7 ms.

### 2.5 Statistical analysis

Results of patient characteristics are presented as mean ± standard deviation or as percentages. Appropriate parametric or non‐parametric statistical tests (Student's t‐test or Wilcoxon signed ranks test for paired data and Student's t‐test or Mann–Whitney U test for unpaired data) were used for continuous variables and chi‐square tests for categorical variables, with the level of significance *p* < 0.05. Statistical analysis was performed using the SPSS Statistical Software Package (IBM SPSS Statistics, version 24).

## 3 RESULTS

### 3.1 Patients

The baseline characteristics of the study patients are summarized in Table 1. Patients had a mean left ventricular ejection fraction of 60 ± 4%, and left ventricular internal diameter in diastole of 49 ± 4 mm. Of the 157 study patients, 105 (67%) had narrow QRS, and 52 (33%) had wide QRS (≥120 ms) of right bundle branch block (RBBB) pattern. A similar proportion of patients with narrow‐QRS vs. RBBB were taking angiotensin‐converting enzyme inhibitors or calcium channel blockers (44% and 52%, respectively). Narrow QRS patients were younger (*p* < 0.05) and had a higher heart rate (*p* < 0.05) compared to RBBB patients. As expected, the RBBB patients had significantly longer QRS duration (*p* < 0.001). Overall, more Medtronic than Abbott pacemakers were implanted (105/52, 73%), but both normal QRS and RBBB patient groups included similar proportions of implanted pacemaker manufacturers (i.e., Medtronic 77% and 65%, respectively, *p* < 0.11). No complications occurred during the implantation procedures.

**TABLE 1:** Patient and procedural characteristics

Variable	Total (*n* = 157)	Narrow QRS (*n* = 105)	RBBB (*n* = 52)	*p*‐value
Age (years)	76 ± 9	75 ± 9	78 ± 8	0.016
Male gender (*n*, %)	92 (58.6)	60 (57.1)	32 (61.5)	NS
Body Mass Index (kg/m ^2^ )	27.6 ± 4.1	27.3 ± 4.1	28.2 ± 4.1	NS
Hypertension	69 (44)	44 (42)	25 (48)	NS
Diabetes	45 (29)	29 (28)	16 (31)	NS
Heart rhythm (*n*, %)				
Sinus bradycardia	124 (79)	91 (87)	33 (63)	0.001
AV conduction disease	33 (21)	14 (13)	19 (37)	0.001
ECG at baseline				
HR (beats/min)	59.1 ± 13.0	61.1 ± 12.8	55.1 ± 12.5	0.007
PR interval (ms)	208 ± 41	209 ± 45	208 ± 45	NS
QRS duration (ms)	114 ± 28	95 ± 11	151 ± 12	<0.001
Pacemaker manufacturer				
Medtronic® Inc./Abbott® Inc., (*n*)	115/42	81/24	34/18	NS
Q‐VS through PSA	56.5 ± 28.4	50.9 ± 24.2	67.8 ± 32.9	<0.001
Q‐VS through Pacemaker	22.5 ± 14.8	18.2 ± 12.8	31.2 ± 14.8	<0.001

*Note*: Values are presented as mean ± SD or as *n* (%). Abbreviations: AV, atrioventricular; HR, heart rate; NS, not significant; Q‐VS = Sensing latency.

### 3.2 Traditional sensing characteristics

All the patients had an R wave amplitude of 9.9 ± 3.7 mV and a slew rate of 2.74 ± 0.94 V/s through the PSA, and a similar R wave of 10.3 ± 3.4 mV through the pacemaker (*p* = 0.46). Overall, there were no differences between narrow QRS vs. RBBB patients in R wave amplitude and slew rate through the PSA (10.1 ± 3.8 vs. 9.9 ± 4.0 mV and 2.66 ± 1.01 vs. 2.67 ± 1.02 V/s, respectively), and in R wave amplitude through the pacemaker (10.5 ± 3.9 vs. 11.1 ± 4.5 mV, respectively). There were also no differences in R wave amplitude or slew rate between the two pacemaker manufacturers when the study patients were considered as a whole group or were analyzed as separate groups of narrow QRS patients vs. RBBB patients (*p* > 0.5 for all comparisons)

### 3.3 Q‐VS through PSA vs. through pacemaker

**FIGURE 3:**
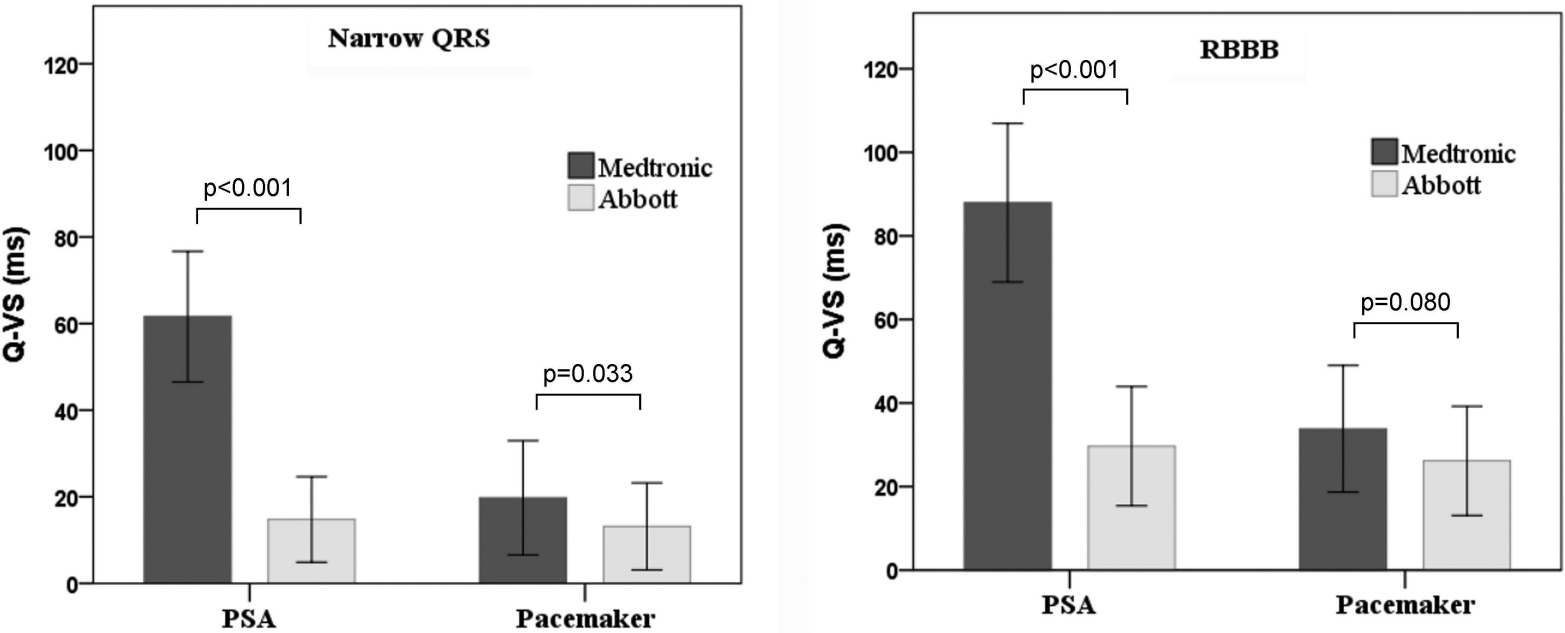
Different RV lead sensing latency (Q‐VS) through PSA and pacemaker in patients with narrow QRS (left panel) or RBBB (right panel). Comparison of RV lead sensing latency between Medtronic and Abbott devices.

Data on the Q‐VS results are presented in Table 1 and Figure 3. All the patients showed greater mean Q‐VS through the PSA as compared through the pacemaker (56.5 ± 28.4 ms [range 0–122 ms] vs. 22.5 ± 14.8 ms [range 0–60 ms] [*p* < 0.0001]). Both narrow QRS and RBBB patients had similarly longer mean Q‐VS through PSA (50.9 ms [range 0–100 ms] and 67.8 ms [range 2–122 ms], respectively) than those values obtained through pacemaker (18.2 ms [range 0–50 ms] and 31.2 ms [range 2–60 ms], respectively) (*p* < 0.0001). Overall, RBBB patients had significantly longer Q‐VS than patients with narrow QRS when assessed either through the PSA or through the pacemaker (*p* < 0.001).

The Q‐VS data according to the manufacturer are shown in Figure 3. For narrow QRS patients, Medtronic's Q‐VS through PSA of 61.5 ± 15.1 ms (range 15–100 ms) was greater as compared with those obtained through pacemaker of 19.7 ± 13.1 ms (range 0–50 ms) (*p* < 0.001). All 81 Medtronic patients with narrow QRS showed shorter Q‐VS through pacemaker than through PSA. Abbott's longer Q‐VS through PSA of 14.7 ± 9.8 (range 0–42 ms) did not change significantly from that obtained through pacemaker of 13.1 ± 10.2 ms, (range 0–37 ms). On 2 of Abbott 24 occasions (8%), the Q‐VS through pacemaker was slightly longer than that through PSA (13.5 ± 7 vs. 9.5 ± 7, *p* = NS). For RBBB patients, similarly, Medtronicʼs Q‐VS through PSA of 87.9 ± 18.9 ms (range 37–122 ms) was greater as compared to those obtained through pacemaker of 33.8 ± 15.1 ms, (range 6–60 ms) (*p* < 0.001), with all patients showing shorter Q‐VS through the pacemaker. Abbottʼs Q‐VS through PSA of 29.7 ± 14.2 ms (range 2–52 ms) did not differ from that obtained through pacemaker of 26.1 ± 13 ms, (range 2–56 ms) (*p* = 0.4), but on 8 of 18 occasions (44%), the Q‐VS through the pacemaker was slightly longer than that obtained through PSA (23.8 ± 16 vs. 22.8 ± 16, *p* = NS). Overall, Medtronic's Q‐VS was longer when compared with that of Abbott in narrow QRS patients through both PSA and pacemaker (*p* < 0.001); RBBB patients had longer Medtronic Q‐VS through PSA, but not through pacemaker (*p* < 0.001 and *p* = 0.08, respectively).

## 4 DISCUSSION

Proper sensing evokes order and meaning to most effective and qualitative pacing and is an essential precondition to promote intrinsic ventricular activity and avoid adverse ventricular pacing.^13^, ^14^, ^15^ As a general rule, the clinician should always keep in mind to judge whether the EGM and marker annotations of the pacemaker agree with what appears to be on ECG. In addition, we should appreciate a time lag for basic pacemaker sensing function determined by the intrinsic EGM detection from the endocardial source to processor interpretation and execution of the operation. More precisely, there is an expected signal propagation delay over the various interconnecting lines in the path of device function (e.g., connection cables and ports, implanted electrode lead, electronic circuitries of PSA, pacemaker, programmer), which is also subject to intrinsic manufacturing design process variations, such as a shift in length or width, or the material. This insight applies to the ECG as well, with the “filter” of intervening tissues and structures that lie between the origination of cardiac activity within the heart and its detection by the electrodes placed on the skin. Thus, the phenomenon of signal propagation delay, either for ECG or for EGM recording, is real, and it is absolutely unavoidable.

During implant, clinicians usually take the device's proper sensing function for granted if the intrinsic EGM signal received by the electrode and transmitted to the PSA sensing circuit has sufficient amplitude and slew rate, whereas often do not take any notice of the signal propagation delay. Of course, as little as possible RV lead sensing latency is desired so as not to confound proper pacemaker operation. Our observation that the QRS consistently preceded the EGM obtained by either PSA or pacemaker concurs with the earlier manifestation of spontaneous activity on ECG.^2^, ^3^ The delayed recording of EGM relative to ECG reflects long RV lead sensing latency, which prevents the intrinsic activity to be interpreted as such by the device and notified on the marker channel as “VS”. Consequently, as the intrinsic activity initially remains out‐of‐sight for the device, the ventricular stimulus may occur and be counted, without causing a subsequent ventricular depolarization on ECG if it is delivered as the ventricle is already contracting.

Focusing on the PSA application of both manufacturers, the EGM signal travels a similar fixed distance in the length of about 4.2 m (PSA cables of approximately 3.7 m and implanted electrode of 0.58 cm) to the terminal unit to be recorded. It is interesting that although for ECG recording the intrinsic ventricular event needs to cover an overall similar cable distance to the skin surface, the PSAs of both manufacturers needed more time for EGM processing to output “VS”. An unexpected finding was also the much shorter RV lead sensing latency of Abbott compared to Medtronic in both patient groups. The notable differences among the patient groups and manufacturers may be explained by differences in QRS waveform and different methods in R point recognition. In any case, these new observations demonstrated substantial RV lead sensing latency which should be taken into consideration for the management of pacemaker programming.

Contrary to the wide‐held view that the implanted pacemaker replicates similar data to those obtained intra‐operatively through the PSA, it had been a pleasure to note much smaller sensing latency through the pacemaker. Specifically, we found through pacemaker mean Q‐VS values of 18.2 ms in Narrow‐QRS patients and 31.2 ms in RBBB patients within a wide range of values to a maximum of 50 ms and 60 ms, respectively. Of note, unlike Medtronic, Abbott showed in 8% of normal QRS patients, and in 44% of RBBB patients, slightly longer Q‐VS through the pacemaker as compared through PSA. The decrease of sensing latency by the implanted pacemaker compared to the PSA measurements was particularly impressive in Medtronic pacemakers of both patient groups, though still longer than in Abbott pacemakers. Possible explanations for the differences seen between Medtronic and Abbott devices include different manufacturing and engineering methods involving possibly different raw materials in lead and generator construction as well as different QRS recognition sensing methods and device band‐pass filters.

### 4.1 Clinical implications in dual‐chamber pacing

Though adjustment of the AV timing cycles intends to ensure optimal mechanical coordination between atrial and ventricular contractions, whether the atrium is sensed or paced, often, it is left programmed in an empirical way or to a predefined manufacture's setting value. Practically, the AV delay starts with an atrial event and allows a precisely timed interval to occur during which the pacemaker seeks activity on the ventricular channel to terminate it. With the sensing latency, the sensed AV interval becomes more prolonged and the ventricular channel waits for more time to alert for potential incoming ventricular activity. After termination of the paced programmed AV delay, the delivered ventricular pulse will not capture the ventricle if it lands on a late conducted intrinsic ventricular beat. The coincidence of a heart's intrinsic beat and a pacemaker spike will result in unnecessary and confusing fusion/pseudofusion which, in addition, may be counted and annotated as a ventricular‐paced event.^6^, ^7^

Reduction of unnecessary ventricular pacing is recommended to be exercised in pacing populations with intact, low‐degree, or intermittent atrioventricular conduction.^14^, ^15^ Ventricular fusion/pseudofusion is not expected to have a major negative impact on the cardiac function but may be misleading by providing false cumulative percentages of VP beats in particular when autointrinsic conduction search algorithms are not available or activated and the clinician uses manual programming and fixed long AV intervals to prolong AV interval. However, even auto‐capture and adaptive AV search algorithms may create a ventricular fusion with resultant unnecessary delivery of backup pulses and a possible increase in pacemaker output.^16^ More specifically, for CRT recipients, a shorter sensed AV interval may be allowed to increase true CRT pacing since inappropriately programmed AV timing intervals have been reported as the most common reason for episodes of sustained loss of CRT capture.^8^

In point of fact, programming a longer AV delay, at least to the value of RV lead electrical delay, may add to the prevention of unnecessary ventricular pacing. This setting is possible since pacemaker manufacturers enable the extension of the AV delay usually in successive steps of 10 ms interval increments. Our findings of wide range RV lead sensing latency interpatient variability suggest possible clinical relevance and individualized AV delay programming, whereby much longer AV interval extensions may be needed particularly in patients with intrinsic RBBB.

### 4.2 Study limitations

Several limitations should be mentioned. First, our Q‐VS data relating to the RV lead placement in the mid‐septal position may not be extrapolated to other RV pacing sites. In addition, other pacemaker manufacturers may provide different RV lead sensing latency results either through PSA or through the implanted pacemaker. Also, there is a question as to whether comparable results might be related to other pacing populations with underlying cardiac disease.

## 5 CONCLUSIONS

The QRS on ECG does not coincide in time with the EGM signal from the RV lead, reflecting an appreciable RV lead electrical delay either through PSA or through the implanted pacemaker. Sensing EGM through PSA incorporates distinctly longer electrical delay compared with the pacemaker generator. RBBB patients exhibit longer RV lead sensing latency than patients with narrow QRS, as do Medtronic devices compared with Abbott devices. By remembering RV lead sensing latency, the extension of the AV delay to the value of RV lead electrical delay contributes to the avoidance of unnecessary ventricular stimuli.

## CONFLICT OF INTEREST

Authors declare no conflict of interests for this article.
